# AI-enabled multimodal neuroimaging for neurotransmitter mapping in normal aging and age-related disease

**DOI:** 10.3389/fnagi.2026.1748671

**Published:** 2026-07-24

**Authors:** Paige Hewitt, Hongsheen Kim, Thomas A. Vida

**Affiliations:** Department of Medical Education, Kirk Kerkorian School of Medicine at UNLV, Las Vegas, NV, United States

**Keywords:** artificial intelligence, cognitive aging, data harmonization, explainable AI, GABA–glutamate balance, mechanistic modeling, multimodal neuroimaging, neurotransmitter mapping

## Abstract

Aging reshapes neurotransmitter systems through nonlinear and region-specific shifts that alter excitatory–inhibitory balance, weaken metabolic coupling, and destabilize large-scale neural networks. PET, MRS, molecular MRI, and optical imaging quantify elements of these trajectories, but modality-specific artifacts, cross-site variability, and limited spatial or temporal resolution fragment mechanistic interpretation. Artificial intelligence now enables the integration of these heterogeneous signals into harmonized, multimodal representations that link molecular alterations to circuit-level dynamics and clinical outcomes. This review demonstrates how AI-enabled fusion can transform neurotransmitter imaging by correcting acquisition bias, enhancing reproducibility, and revealing hidden dependencies among GABAergic, glutamatergic, cholinergic, dopaminergic, and serotonergic systems. We advance three hypotheses: that conserved neurotransmitter coupling patterns distinguish healthy from pathological aging; that excitatory–inhibitory reorganization reflects compensatory signaling rather than linear degeneration; and that AI-driven multimodal integration can generate mechanistic predictors of cognitive, affective, and motor decline. By embedding PET-, MRS-, and MRI-derived features into explainable architectures, AI models can identify early neurochemical inflection points, stratify individuals by neurotransmitter vulnerability, and support precision diagnostics. AI-enabled multimodal neuroimaging, therefore, establishes a foundation for mechanistic neuroscience in aging, reframing neurochemical decline as a dynamic interplay between adaptation and vulnerability rather than a uniform trajectory of loss.

## 1 Introduction

### 1.1 Neurotransmitters in aging and disease

Neurotransmitters play a crucial role in regulating synaptic signaling and neuronal network dynamics, making them essential to understanding both healthy brain aging and the pathogenesis of age-related neurological and psychiatric disorders (Kringelbach et al., 2020; Hansen et al., 2022). Across the lifespan, these systems follow nonlinear and region-specific trajectories that reshape excitatory–inhibitory balance (Rmus et al., 2023), alter metabolic coupling between neurons and glia (Ling et al., 2024), and modify the computational capacity of cortical and subcortical circuits (Traxler et al., 2021). MRS and PET reveal age-related declines in GABA and glutamate within the anterior cingulate, hippocampus, and striatum, accompanied by progressive reductions in cholinergic tone and more subtle dopaminergic and serotonergic shifts (Zahr et al., 2008; Huang et al., 2016; Porges et al., 2017; Zeydan and Kantarci, 2021; Lee and Kim, 2022; Dahl et al., 2023; Schreiner et al., 2024; Yoo et al., 2024). These changes influence working memory, executive control, and episodic memory long before clinical impairment emerges, positioning neurotransmitter dynamics as early determinants of cognitive aging rather than downstream consequences of structural decline (Jin and Cai, 2023; Andersson et al., 2025). Understanding these neurochemical trajectories, therefore, provides a mechanistic foundation for distinguishing adaptive remodeling from pathological degeneration across aging brains (Bunzeck et al., 2024). These dynamics manifest through distinct neurotransmitter systems, each of which follows a characteristic aging trajectory that shapes cognition and vulnerability to disease.

#### 1.1.1 GABAergic and glutamatergic systems

Among all neurotransmitters, the GABAergic and glutamatergic systems exhibit the most pronounced age-related alterations and provide the most robust evidence for region-specific decline (Porges et al., 2021; Andersson et al., 2025; Treacy et al., 2025). Nonlinear, region-specific changes in neurotransmitter levels occur during normal aging and in age-related neurological and psychiatric conditions (Rozycka et al., 2019; Ebrahimi et al., 2025; Pecsok et al., 2025). Magnetic resonance spectroscopy and PET persistently reveal decreases in GABA, the primary inhibitory neurotransmitter in the human nervous system, and glutamate, the primary excitatory neurotransmitter, in the anterior cingulate, hippocampus, and striatum as well as a gradual decrease in acetylcholine of about 3.7% per decade (Kuhl et al., 1996; Huang et al., 2016; Porges et al., 2017). After controlling for atrophy, this reduction in GABA persists, indicating a genuine decrease in GABA concentration in brain tissue rather than a consequence of cortical atrophy (Porges et al., 2017). GABA concentrations are also associated with cognitive performance (Porges et al., 2017; Chamberlain et al., 2021), which remains significant even after controlling for factors such as age and education (Porges et al., 2017; Schreiner et al., 2024). Simultaneous decreases in glutamate also occur (Zeydan and Kantarci, 2021). Unchanged GABA concentrations accompanied by decreased glutamate concentrations occur in patients with Alzheimer’s disease (Huang et al., 2016) and increased GABA/glutamate ratios are associated with higher amyloid-beta levels in healthy, elderly patients (Schreiner et al., 2024).

#### 1.1.2 Cholinergic and dopaminergic pathways

Cholinergic and dopaminergic pathways also exhibit distinct aging patterns, and their combined disruption directly contributes to cognitive decline and network instability (Hsu et al., 2021; Russell et al., 2025; Zaccone et al., 2024). Significant cholinergic and glutamatergic losses take place in the hippocampus, posterior cingulate, and dorsolateral prefrontal cortex in Alzheimer’s disease (Riese et al., 2015; Zaccone et al., 2024). Widespread loss of cholinergic terminals in the neocortex and hippocampus occurs in early-onset disease (Roy et al., 2016). In late-onset disease, reductions are usually limited to the temporal cortex and hippocampus. Terminal deficits are extensive in patients with severe dementia (Kuhl et al., 1996). Although a reduction in GABA typically occurs, paradoxical elevations in GABA are also reported in patients with advanced Alzheimer’s. Although results vary across cohorts, cerebral MRS and fMRI reveal a negative correlation between GABA levels and cognitive scores (Dhanjal et al., 2014). These converging, paradoxical findings underscore the need for more precise, multimodal imaging to better characterize the changing GABA concentrations in Alzheimer’s disease.

In Parkinson’s disease, early dopaminergic depletion occurs in the striatum before clinical symptoms present. Individuals with minimal cognitive changes frequently present with dopaminergic denervation in the caudate nucleus, and the magnitude of this change is further increased in patients with more severe cognitive impairments (Bohnen et al., 2015). Additionally, patients with dementia with Lewy bodies and Parkinson’s disease dementia demonstrate both dopaminergic and cholinergic deficits. These cholinergic deficits appear to play an important role in the development of dementia in addition to motor symptoms (Klein et al., 2010). As dopamine levels decrease in the caudate nucleus, executive, visuospatial, and memory scores also decrease (Yoo et al., 2024). As dopaminergic depletion occurs in the putamen, rigidity and bradykinesia scores increase (Yoo et al., 2024). The literature across species also suggests that degeneration of the locus coeruleus, a structure involved in catecholaminergic modulation, decreases dopaminergic and noradrenergic modulation of hippocampal plasticity, leading to memory decline (Dahl et al., 2023). Parkinson’s disease is also characterized by cortical cholinergic deficits and decreased glutamate levels (Buard et al., 2022). Proton Magnetic Resonance Spectroscopy demonstrates disrupted glutamatergic metabolism in the prefrontal cortex, reflected by reduced glutamate-to-creatine resonance ratios in patients with cognitive symptoms (Buard et al., 2022). Although cortical cholinergic denervation is less common in Parkinson’s disease patients with mild cognitive impairment, it becomes very frequent in patients with more severe cognitive deficits (Bohnen et al., 2015). A cross-sectional study also links the degeneration of the posterior basal forebrain in Parkinson’s disease to changes in acetylcholinesterase activity. Both PET and MRI cholinergic imaging markers are associated independently with cognitive deficits in multiple domains in patients with Parkinson’s disease without dementia (Schumacher et al., 2023). The multimodal integration of PET and MRI, combined with AI, can integrate these independent findings to provide a broader, translational framework that links neurotransmitter loss to measurable clinical outcomes.

#### 1.1.3 Serotonergic modulation and late-life depression

Serotonergic modulation adds a third layer of neurochemical vulnerability, especially in late-life depression, where inhibitory–excitatory imbalance intersects with monoaminergic decline (Khoodoruth et al., 2023; Zhao et al., 2023; Bremshey et al., 2024). The serotonergic pathway affects appetite, sleep, learning, memory, temperature, mood, muscle contraction, and both cardiovascular and endocrine function (Nikhra, 2017). Serotonin receptors and transporters decrease with age, and binding capacity of serotonin transporters in the thalamus and midbrain also declines (Lee and Kim, 2022). Late-life depression can lead to disruptions in serotonergic and GABAergic tone. Multimodal PET–MRS integration demonstrates that decreased transporter binding and altered inhibitory tone co-occur with mood and cognitive deterioration (Smith et al., 2021b). PET imaging with radiotracers for beta-amyloid in patients diagnosed with late-life depression and healthy controls found that higher levels of beta-amyloid in the temporal, parietal, and occipital cortices is associated with lower levels of serotonin in the putamen, thalamus, amygdala, hippocampus, and raphe nuclei compared to controls (Smith et al., 2021b). Serotonergic receptors and transporters also decline in Alzheimer’s disease (Ouchi et al., 2009). Patients with earlier-stage Alzheimer’s disease demonstrate a significant reduction in serotonin transporter marker binding, particularly in the striatum, regardless of their depression status (Ouchi et al., 2009).

### 1.2 Mechanistic gaps in current neuroimaging approaches

**Figure 1:**
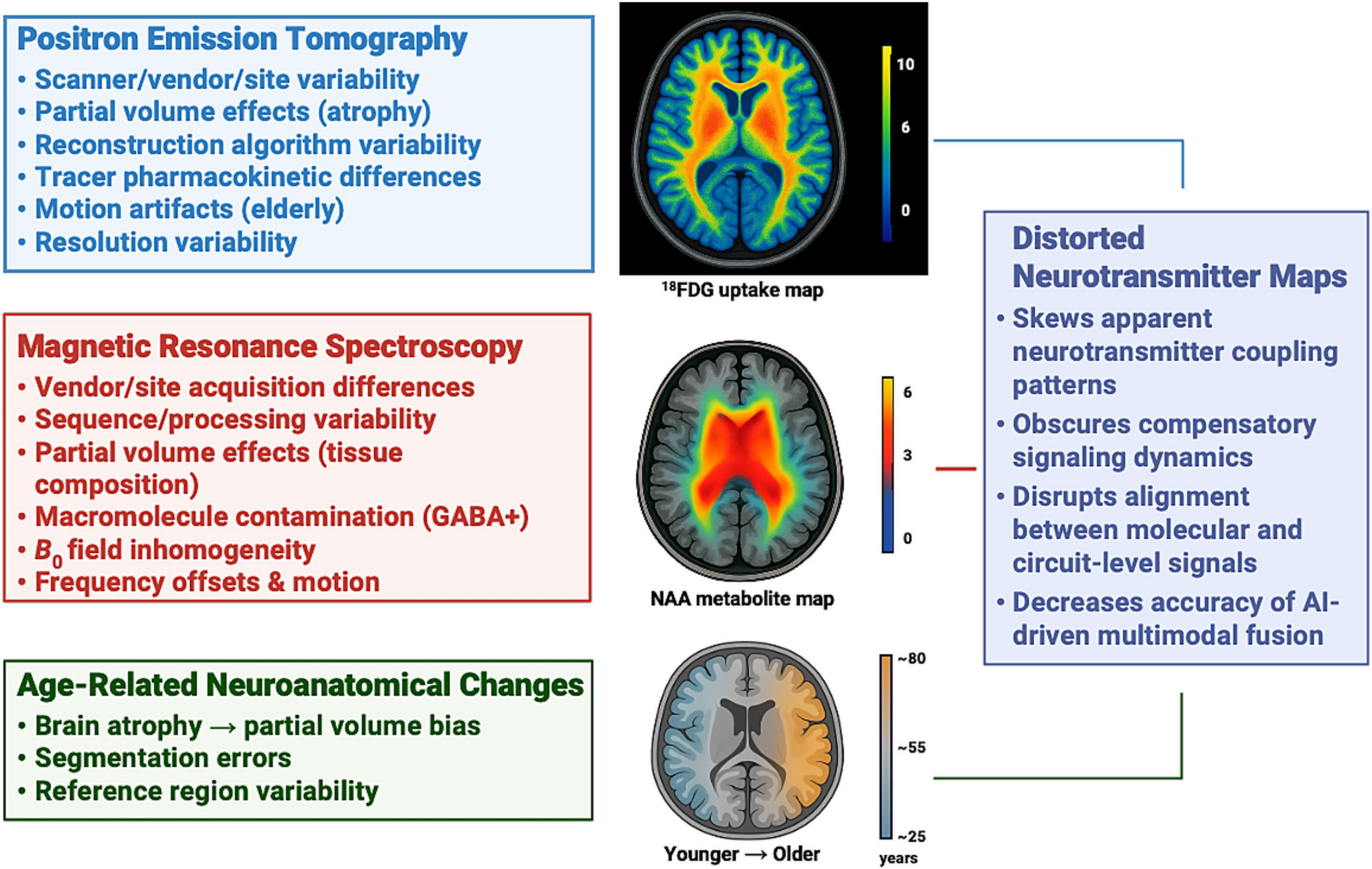
Sources of imaging artifacts in PET and MRS studies of aging brains. Sources of distortion in neurotransmitter imaging and their impact on mechanistic interpretation. This figure illustrates how modality-specific artifacts and biological variability distort quantitative neurotransmitter maps in aging and neurodegeneration. Positron emission tomography (PET) (upper left, blue) exhibits scanner and vendor variability, partial volume effects due to cortical atrophy, reconstruction algorithm differences, tracer pharmacokinetic variability, and motion artifacts in elderly populations, all of which degrade spatial fidelity of molecular signals. Magnetic resonance spectroscopy (MRS) (center left, red) suffers from inter-site acquisition variability, sequence and processing inconsistencies, macromolecule contamination (GABA^+^), tissue composition–driven partial volume effects, field inhomogeneity (B₀), and frequency offsets caused by motion. Age-related neuroanatomical changes (lower left, green) such as cortical thinning, segmentation error, and reference-region instability compound these modality-specific distortions. Together, these factors produce distorted neurotransmitter maps (right, violet) that skew apparent neurotransmitter coupling patterns, obscure compensatory signaling dynamics, and disrupt alignment between molecular and circuit-level measures. Such distortions reduce the accuracy of AI-driven multimodal fusion models designed to infer causal relationships between neurotransmitter imbalance, network dysfunction, and cognitive decline. Representative brain images illustrate PET-derived glucose metabolism (^18^FDG uptake map), MRS-detected N-acetylaspartate distribution (NAA metabolite map), and age-related cortical thinning (structural MRI, younger → older gradient). Approximate scale and acquisition parameters: PET spatial resolution ≈ 3–5 mm full width at half maximum (FWHM); signal expressed as standardized uptake value ratio (SUVr), dynamic range 0–10. MRS voxel size ≈ 20–30 mm^3^; spectral frequency resolution 0.02–0.04 ppm; metabolite concentrations approximated in institutional units (NAA ≈ 3.0 IU; GABA ≈ 1.0 IU). Cortical thickness gradient represents ages 25–80 years with range 1.8–2.7 mm. All figure panels are AI-generated conceptual illustrations created for explanatory purposes only and do not depict real PET, MRS, or MRI data.

Across neurological and psychiatric conditions, cognition is strongly correlated with changes in neurotransmitters. Decreases in acetylcholine and dopamine correlate with executive dysfunction, reduced GABA in the frontal cortex explains variance in global cognition, and reduced glutamate correlates with episodic memory impairment (Matsuoka et al., 2024). A decline in GABA and glutamate can usually be detected in aging and cognitive impairment prior to onset of clinical symptoms (Huang et al., 2016). Although these trajectories reveal converging neurochemical themes across disorders, current imaging modalities cannot resolve how these changes interact mechanistically across scales. Despite significant progress in molecular and metabolic imaging, today’s modalities can only capture fragmented representations of the processes involved in neurotransmission. Current techniques are limited to providing a static snapshot rather than a multimodal, dynamic model of neural communication and signaling. PET is molecularly specific and measures receptor and transporter binding potential (Sedvall et al., 1990; Tuominen et al., 2013). Radiotracers such as [^18^F]FEOBV for vesicular acetylcholine transporters (Horsager et al., 2022; Kanel et al., 2022), [^18^F]-XTRA for α4β2 nicotinic transporters (Coughlin et al., 2018), [^11^C]MADAM for serotonin transporters (Lundberg et al., 2006), and [^18^F]fluorodopa for dopaminergic pathways (Keir et al., 2024; Stormezand et al., 2024) can uncover molecularly specific findings. Although PET can serve as an indirect proxy for synaptic transmission, it is unable to image transient flux and variation in neurotransmitter systems associated with receptor plasticity over time (Schain et al., 2012). Variations in the binding potential and affinity of neurotransmitters and receptors can also misrepresent true receptor density and obscure downstream signaling effects that are crucial for mechanistic interpretation (Finnema et al., 2015; Aghourian et al., 2016). MRS can quantify bulk metabolite concentrations; however, it lacks the spatial granularity required to resolve neurotransmitter compartmentalization between neurons and glia (Simmonite et al., 2019; Cuypers et al., 2021). Due to this missing element, MRS is unable to map shifts in metabolite ratios onto excitatory-inhibitory balances and dynamics. Additionally, molecular MRI and optical imaging have insufficient temporal resolution, which restricts the application of cellular mechanisms to the scale of whole-brain circuitry (Marvin et al., 2013; Beyene, 2019). Despite recent advances in neuroimaging, the limitations of current modalities reveal a critical mechanistic gap. These methods are unable to link to changes in larger-scale networks and behavior over time. Current methods can reveal where neurotransmitter levels change; however, they cannot show how these changes drive neural communication, computation, degeneration, and adaptation. Additionally, inter-modality inconsistencies, such as differences in spatial scale, signal types, and noise structure, preclude direct comparisons and the inference or suggestion of causal relationships. Harmonized, longitudinal models are needed to differentiate between compensatory and pathological neurotransmitter changes and adaptations (see Figure 1).

### 1.3 Hypotheses and conceptual framework

These limitations form the rationale for an AI-centered framework that can link molecular alterations to circuit-level signaling and behavioral outcomes. The gaps in today’s neuroimaging methods are the central motivation for integrating artificial intelligence. AI offers the potential to enhance neuroimaging by fusing molecular, metabolic, and structural data to construct mechanistic trajectories, rather than static correlates of normal aging and pathological changes. Three complementary hypotheses guide this review. First, the *Coupling Pattern Hypothesis* states that AI-enabled multimodal neuroimaging can reveal conserved, system-level neurotransmitter coupling patterns that distinguish normal aging from age-related pathology, thereby providing a mechanistic framework for early diagnosis and therapeutic targeting. Second, the *Compensatory Signaling Hypothesis* proposes that the nonlinear, region-specific changes in excitatory and inhibitory signaling observed across aging and neurodegeneration reflect adaptive—rather than purely degenerative—processes, and that AI models trained on longitudinal, multimodal data can quantify these compensatory dynamics. Third, the *Mechanistic Integration Hypothesis* posits that integrating PET, MRS, MRI, and optical imaging into AI architectures transforms neuroimaging from a descriptive modality into a mechanistic science, enabling causal inference linking molecular decline to network dysfunction and clinical outcomes. These hypotheses form the foundation for the subsequent sections, which examine biological mechanisms, technical barriers, and AI-driven solutions within a unified mechanistic–translational framework. Together, they describe a progressive logic: mapping conserved neurotransmitter patterns (*Coupling Pattern Hypothesis*), interpreting adaptive signaling dynamics (*Compensatory Signaling Hypothesis*), and integrating multimodal data to model causality across molecular, circuit, and clinical scales (*Mechanistic Integration Hypothesis*).

This review provides a mechanism-oriented framework for interpreting neurotransmitter imaging in normal aging and age-related disease. We distinguish three levels of evidence. First, we synthesize established findings from PET, MRS, MRI, and related imaging modalities that quantify neurotransmitter receptors, transporters, metabolite concentrations, and imaging limitations relevant to aging and neurodegeneration (Finnema et al., 2015; Huang et al., 2016; Beyene, 2019; Simmonite et al., 2019; Tiepolt et al., 2022; Okkels et al., 2023; Schreiner et al., 2024). Second, we evaluate validated and emerging AI methods for reconstruction, denoising, harmonization, multimodal fusion, prediction, and interpretability. Third, we propose forward-looking hypotheses that require prospective testing, external validation, and explicit criteria for falsification. This structure shifts the review from descriptive aggregation toward testable mechanistic synthesis. We use the Coupling Pattern, Compensatory Signaling, and Mechanistic Integration hypotheses to define measurable predictions, validation requirements, falsification conditions, and translational priorities for AI-enabled neurotransmitter mapping. Table 1 operationalizes these hypotheses by linking each conceptual claim to measurable variables, AI methods, success criteria, falsification conditions, and translational relevance.

**Table 1:** Operationalizing the three hypotheses for AI-enabled neurotransmitter mapping.

Hypothesis	Biological mechanism	Measurable variables	AI methodological approach	Quantitative success criteria	Falsification conditions	Translational relevance
Coupling pattern hypothesis	Neurotransmitter systems co-vary across molecular, metabolic, and circuit scales during aging and disease	PET receptor/transporter measures; MRS GABA/Glu/Glx; MRI/fMRI connectivity; cognitive outcomes	Multimodal fusion, normative modeling, graph neural networks, cross-site harmonization	Multimodal coupling patterns improve external-cohort prediction beyond unimodal models	Coupling patterns disappear after harmonization or fail external validation	Early stratification of healthy vs. pathological aging
Compensatory signaling hypothesis	Nonlinear excitatory–inhibitory changes initially stabilize circuits before becoming maladaptive	Longitudinal GABA/Glu/Glx; PET receptor availability; network connectivity; cognitive/motor/affective trajectories	Longitudinal modeling, trajectory clustering, uncertainty-aware prediction	Models identify inflection points that predict preserved function followed by decline	Neurotransmitter shifts show no temporal relation to clinical or network outcomes	Therapy monitoring and detection of adaptive vs. maladaptive response
Mechanistic integration hypothesis	Integrated molecular, metabolic, structural, and functional signals explain clinical decline better than isolated measures	PET, MRS, MRI/fMRI, DTI, clinical endpoints	Hybrid AI-mechanistic models, virtual brain models, digital twins, XAI	Fused models improve prediction, calibration, interpretability, and biological plausibility	Fusion adds no value beyond unimodal imaging or standard clinical variables	Precision diagnostics, individualized monitoring, and model-guided intervention

## 2 Imaging modalities capture neurochemistry but fail to resolve system-level dynamics

Advances in neuroimaging enable the non-invasive examination of human neurotransmitter systems; however, each modality has specific limitations and offers only a narrow view of the broader clinical picture.

### 2.1 PET radiotracers map neurotransmitters with high sensitivity but limited mechanistic resolution

#### 2.1.1 Radiotracers enable molecular-specific imaging but constrain dynamic interpretation

Positron emission tomography (PET) enables molecular mapping of neurotransmitter systems and remains the gold standard for receptor quantification (Tuominen et al., 2013). Tracers targeting acetylcholine, dopamine, serotonin, glutamate, and GABA provide visualizations of distributed receptor networks with millimeter-scale precision (Okkels et al., 2023). Radiotracers enable the visualization of dopaminergic, cholinergic, and serotonergic systems. Acetylcholine tracers, such as [^18^F]FEOBV and [^18^F]VAT, delineate vesicular transporter density in cortical and subcortical regions and expose early cholinergic vulnerability in Alzheimer’s disease and Lewy Body Dementia (Tiepolt et al., 2022). PET and SPECT were the first imaging methods to evaluate dopamine in the human brain. Several dopamine D2-like receptor radioligands have been tested for sensitivity to endogenous dopamine using ^11^C- or ^18^F-labeled radioligands in PET in both animals and humans (Finnema et al., 2015). Additionally, serotonin transporter tracers such as [^11^C]MADAM offer resolutions between 1.5–4.5 millimeters (Schain et al., 2012) and reveal mood-related serotonergic dysregulation (Schain et al., 2012; Finnema et al., 2015). PET radiotracers are less well established for glutamate and GABA, primarily due to factors such as sensitivity to receptor density, small effect sizes, and allosteric interactions (Finnema et al., 2015). Reports on glutamate and GABA neurotransmitters also lack specific resolution details (Miyake et al., 2011; Mu et al., 2020). Although PET provides molecular specificity, its drawbacks include radiation exposure, high cost, and limited spatial resolution.

#### 2.1.2 Physics, kinetics, and partial volume effects restrict PET’s mechanistic value

Variations in reconstruction algorithms, reference regions, and tracer kinetics introduce spatial biases that fragment quantitative consistency across cohorts (O’Donnell et al., 2024). According to our first hypothesis, AI-enabled harmonization of PET data can resolve biases by merging receptor maps across tracers and scanners, revealing conserved neurotransmitter coupling patterns that can differentiate normal aging from pathological aging. With AI-enabled harmonization, PET can serve not only as a molecular imaging tool but also as a foundation for mechanistic modeling. Machine learning can transform static receptor maps into predictive biomarkers of decline on a systemic level.

### 2.2 Magnetic resonance spectroscopy (MRS) and molecular probes measure neurochemicals at different scales but do not offer complete neurotransmitter maps

Magnetic resonance spectroscopy (MRS) is a radiation-free alternative imaging modality that can measure neurochemicals such as GABA and glutamate. Techniques such as MEGA-PRESS at 3 Tesla and high-field spectroscopy imaging at 7 Tesla are used (Simmonite et al., 2019; Schreiner et al., 2024). MEGA-PRESS is an MRS method that can separate GABA from other metabolites, and it has been used to examine GABA levels in the brains of both healthy individuals and patients with neurological disorders (Huang et al., 2016). Voxel sizes of about 20–30 millimeters are used in these techniques and they are able to measure both cortical and subcortical levels (Simmonite et al., 2019). Technical advances in spectral editing have also improved its precision. However, spectral overlap with metabolites such as glutamate and glutamine reduces interpretability, especially in older populations who have lower signal-to-noise ratios (Simmonite et al., 2019). Molecular MRI probes and fluorescence-based imaging have the potential to expand to synaptic and subcellular scales. Techniques such as two-photon microscopy using iGluSnDR, fluorescence imaging (EOS), and near-infrared catecholamine nanosensors (nIRCat) can achieve spatial resolutions on scales of 0.25 micrometer to several micrometers and temporal resolution of milliseconds to seconds (Namiki et al., 2007; Marvin et al., 2013; Beyene, 2019). These techniques can map neurotransmitter dynamics in animal models at the cellular levels; however, applications in humans have not been reported. The imaging methods mentioned above can capture snapshots of neurotransmitter dynamics, but none are able to provide comprehensive, quantitative, and temporally resolved maps of neurotransmitter signaling in the aging or diseased brain (Table 2).

**Table 2:** Imaging modalities for neurotransmitter mapping: targets, resolutions, and limitations.

Modality	Primary targets	Resolution	Strengths	Limitations
PET (positron emission tomography)	Dopamine (D1, D2/D3, DAT); Serotonin (5-HT1A, 5-HT2A, SERT); Acetylcholine (AChE, VAChT); GABA-A; Glutamate receptors	High molecular specificity; spatial resolution ~4–6 mm	Quantitative receptor/transporter mapping; sensitive to subtle changes; established clinical use	Radiation exposure; high cost; short tracer half-life; limited accessibility
MRS (magnetic resonance spectroscopy)	GABA, Glutamate (Glu, Glx), NAA, Creatine, Choline	Voxel size ~1–3 cm^3^; limited temporal resolution	Non-invasive; radiation-free; widely available; quantifies metabolites in vivo	Low SNR; spectral overlap (e.g., Glu/Glx); motion artifacts; limited sensitivity to small changes
Molecular MRI	Manganese-based probes; CEST agents; receptor-targeted ligands	Variable; potentially high spatial resolution depending on probe	Combines molecular and anatomical sensitivity; non-ionizing	Experimental; limited validated probes in humans; low sensitivity; regulatory barriers
Optical/fluorescence imaging	Synaptic proteins; receptor subunits; calcium signaling	Micron-level spatial and high temporal resolution	Circuit- and cell-level specificity; dynamic monitoring of neurotransmitter activity	Primarily preclinical; invasive labeling; poor translation to humans

### 2.3 Modalities provide complementary but fragmented neurochemical signals

PET offers high molecular specificity with millimeter-level resolution but is limited by radiation exposure, high cost, and tracer variability (Chaudhari et al., 2021; Duclos et al., 2021; Akamatsu et al., 2023). MRS enables non-invasive measurement of GABA and glutamate but faces challenges such as spectral overlap, low signal-to-noise ratio, and large voxel sizes (Spurny et al., 2019; Otaduy, 2022; Koolschijn et al., 2023). Molecular MRI and optical/fluorescence techniques provide high spatial and temporal resolution in preclinical models, although their use in humans remains limited (Sosnovik and Weissleder, 2007; Farkas, 2021; Schouw et al., 2021). Collectively, these methods illustrate the trade-offs among resolution, sensitivity, and translational feasibility (Kang et al., 2023).

### 2.4 AI integrates modalities and reveals mechanistic patterns

**Figure 2:**
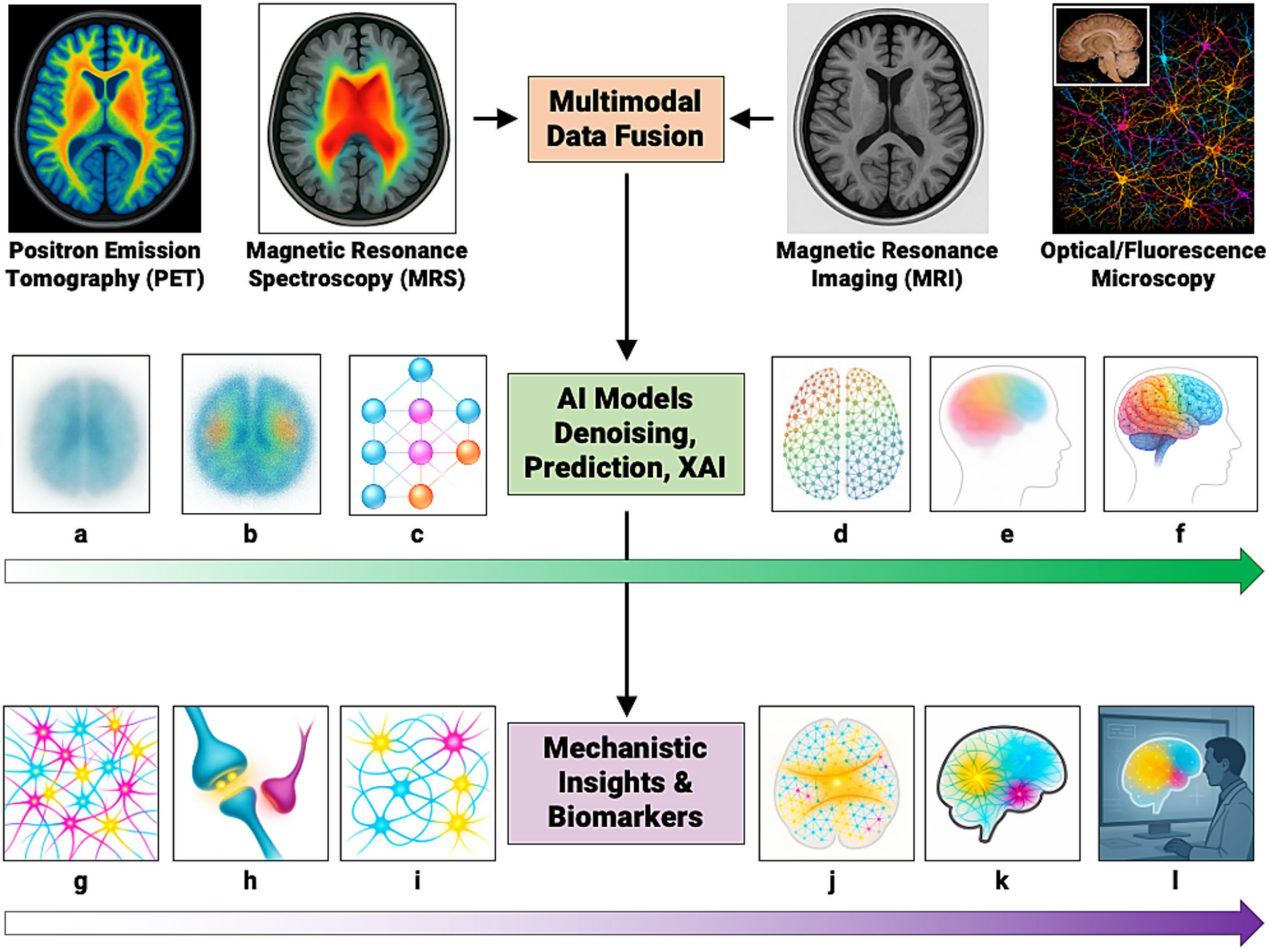
AI-enabled multimodal fusion and mechanistic inference pipeline for neurotransmitter mapping in normal aging and neurodegenerative disease. This schematic visualizes the progressive integration of biological and computational domains within the NeuroMap AI framework, tracing the epistemic transition from raw neuroimaging data to clinically interpretable biomarkers. Multimodal neuroimaging inputs—positron emission tomography (PET), magnetic resonance spectroscopy (MRS), structural magnetic resonance imaging (MRI), and optical/fluorescence microscopy—capture complementary molecular, metabolic, and structural signals across spatial scales. These heterogeneous inputs converge in the multimodal data fusion hub, where cross-modal alignment reconciles discrepancies in spatial resolution, signal contrast, and measurement bias to form a unified analytical substrate. AI models execute denoising, predictive mapping, and explainable inference (XAI), transforming diffuse, low-resolution signals **(a,b)** into structured, interpretable network-level representations **(c–f)** that reflect a trajectory of increasing regularity and abstraction. The mechanistic inference layer links biological signal generation to emergent clinical meaning. Neuronal microcircuits initiate spontaneous activity **(g)**, which self-organizes through patterned synaptic interactions **(h)** and consolidates into mesoscale motifs **(i)**. AI-driven harmonization integrates these motifs into dynamic, system-level representations of neurotransmitter activity **(j)**, from which distinct biomarker domains emerge across the cortical landscape **(k)**. The final panel **(l)** illustrates human–AI interpretive synergy, where translational insights derived from mechanistic models guide diagnostic reasoning and therapeutic decision-making. Collectively, the figure depicts the continuum from raw multimodal acquisition to AI-mediated mechanistic insight, demonstrating how NeuroMap AI unifies molecular, cellular, and systems-level representations of neurotransmission across the spectrum of aging and neurodegeneration.

Artificial intelligence is driving a paradigm shift in processing, integrating, and interpreting imaging data, thereby advancing our understanding of aging and disease mechanisms across multiple scales (Iqbal et al., 2021; Pain et al., 2022) (see Figure 2). Deep learning models enhance raw data quality by denoising PET and MRS images and correcting motion artifacts common in older participants (Gong et al., 2019; Wang J. et al., 2023). Convolutional neural networks (such as 3D Dense-U-Net, ScaleNet), generative adversarial networks (cGAN, 3D-StyleGAN), and Pareto-optimized architectures aid in denoising, reconstruction, and simulation of PET scans for neurotransmitter mapping (Liu et al., 2018; Lee et al., 2019; Pain et al., 2022; Odusami et al., 2023a; Weyts et al., 2024). 3D Dense-U-Net models can impute tau PET images using cross-modality imaging inputs (Lee et al., 2019). These models generate tau PET images based on patterns such as hypometabolism, cerebral atrophy, and amyloid burden (Lee et al., 2019). Two convolutional neural networks, ScaleNet and HighRes3DNet, along with a conditional GAN (cGAN), were trained to help eliminate biases in quantifying beta-amyloid from amyloid-PET scans caused by variations in the tracer’s nonspecific (NS) binding due to cerebrovascular disease. These neural networks and GAN were trained to map structural MR images to NS PET images, with NS estimates subtracted from the standardized uptake value ratio (SUVr) to specifically assess amyloid load (Liu et al., 2021). Multimodal ScaleNet outperformed other networks in quantifying NS content in cortical gray matter, with a mean relative error of less than 2%. The resulting beta-amyloid measurements showed increases of up to 67% in relation to cognitive and functional metrics. However, these networks tended to underestimate NS uptake in some white matter regions (Liu et al., 2021). GANs have also demonstrated the ability to accurately estimate the average global cortical-to-cerebellum SUVR via a linear regression model from the latent space (Bossa et al., 2024). This network generated synthetic PET trajectories and predicted beta-amyloid changes over a four-year period, comparing them to actual progression, highlighting the potential of generative AI to support statistical prediction, disease progression modeling, and simulation of patient outcomes (Bossa et al., 2024). These approaches improve the accuracy and interpretability of neurotransmitter imaging by reducing inter-site variability and enhancing cross-scanner consistency. This trajectory supports the *Mechanistic Integration Hypothesis*: AI-enabled multimodal frameworks transform neuroimaging into a mechanistic science, map molecular decline onto network dysfunction, and link these patterns to clinical outcomes.

Additionally, AI enables the fusion of multimodal data. This fusion combines data from PET-derived receptor densities, MRS-derived neurochemical concentrations, and anatomical context from structural MRI during analysis, providing a more comprehensive clinical picture. Fusion approaches can combine data from multiple modalities using early feature fusion, attention-driven models, and transformer-based methods (Pan and Wang, 2022; Odusami et al., 2023a; Martinez-Murcia et al., 2024). These approaches can reveal coordinated shifts across multiple neurotransmitter systems, including concurrent dopaminergic decline and glutamate-driven hyperexcitability. Mechanistic models of neurotransmitter interactions propose this pattern (de Bartolomeis et al., 2022), and early multimodal PET–MRS evidence supports this prediction (Howes et al., 2020). The use of proton MRS to estimate anterior cingulate cortex (ACC) glutamate levels and ^18^F-DOPA PET to measure dopamine synthesis capacity in the striatum revealed an inverse correlation between ACC glutamate and striatal dopamine in some subjects (Jauhar et al., 2018). This finding is consistent with models demonstrating that cortical glutamatergic drive plays a role in regulating subcortical dopamine synthesis and levels. However, the effect varied between subgroups, and a limitation of MRS in this context is that MRS-derived glutamate reflects both intra- and extra-synaptic pools, thereby limiting specificity (Jauhar et al., 2018). Additionally, combining PET/MRI with PET-measured D2/D3 receptor availability and MRS-measured ACC glutamate and glutamine levels reveals a negative association between higher ACC glutamate and glutamine levels and D2/D3 receptor availability (Rogeau et al., 2022). However, PET and MRS did not find significant associations between hippocampal glutamate and striatal dopamine (Howes et al., 2020). This shows that fusion methods do not always reveal predicted dopamine-glutamate coupling due to factors and limitations such as population heterogeneity, measurement noise, and spatial mismatch. AI-enabled strategies—such as targeted anatomical pairing, longitudinal acquisition, denoising and signal-boosting methods, simultaneous PET–MRS mapping, and interaction or moderation modeling—can delineate the dopamine–glutamate relationship with greater precision.

A cross-modal transformer generative adversarial network (CT-GAN) provides one example of a fusion framework. CT-GAN integrates functional information from resting-state fMRI with structural information from diffusion tensor imaging to generate multimodal connectivity profiles. These profiles predict Alzheimer’s disease trajectories with high internal consistency and match patterns documented in clinical Alzheimer’s disease datasets (Pan and Wang, 2022). Graph Neural Networks (GNNs) treat brain regions as nodes and interactions as edges, enabling the capture of both functional and structural connectivity patterns (Zhao et al., 2023; Ling et al., 2025). A GNN with a Regional Radiomics Similarity Network (R2SN) framework, integrated with transformer-based attention mechanisms, can construct a metabolic network that preserves pathological specificity while quantifying spatial synergistic patterns of microscopic metabolic heterogeneity (Ling et al., 2025).

AI systems can identify early biomarkers, predict and model progression trajectories, and stratify patients based on neurotransmitter vulnerability profiles by extracting latent features and predictive signatures. This capability could shift imaging from a purely descriptive tool to one with hypothesis-generating power, revealing connections between molecular changes and clinical outcomes. AI-driven multimodal integration positions neurotransmitter imaging at the crossroads of molecular and mechanistic understanding and translational therapeutics, enabling earlier intervention and more accurate monitoring of aging and neurodegeneration. These mechanistic insights reflect the trajectory predicted by the *Compensatory Signaling* and *Mechanistic Integration* hypotheses, which anticipate that AI-enabled fusion will clarify cross-system dynamics and enable clinically meaningful predictions of aging and neurodegeneration.

Testing these hypotheses requires AI models that fuse PET-, MRS-, and MRI-derived features into unified neurotransmitter coupling maps and evaluate whether the resulting patterns remain stable across cohorts, scanners, and disease states. Multimodal integration of PET and MRS demonstrates that presynaptic density correlates with cortical glutamate concentration, establishing the feasibility of quantifying neurotransmitter coupling across modalities (Onwordi et al., 2025). Integration of PET-derived receptor density maps across multiple neurotransmitter systems, together with functional MRI connectivity, reveals that receptor architecture constrains large-scale network dynamics (Hansen et al., 2022). Deep-learning frameworks that combine PET and MRI link glucose metabolism with oxygen utilization in aging brains, illustrating the capacity of AI-driven fusion models to map metabolic dependencies (Wang L. et al., 2023). Collectively, these findings provide empirical and methodological grounding for testing the *Coupling Pattern Hypothesis*, which posits that conserved excitatory–inhibitory relationships distinguish healthy aging from early neurodegeneration, and the *Mechanistic Integration Hypothesis*, which proposes that fused molecular, metabolic, and structural representations enable causal inference, predict longitudinal decline, and identify inflection points in neurotransmitter imbalance more accurately than unimodal analyses. Examining the biological mechanisms that generate these coupling patterns and signaling transitions builds on this foundation.

## 3 Biological–mechanistic hypotheses AI can address

### 3.1 Aging drives region-specific neurotransmitter imbalance

Normal aging alters neurotransmitter systems in region-specific and nonlinear ways. Neuroimaging quantifies consistent declines in GABA and glutamate concentrations within the anterior cingulate, hippocampus, and striatum (Zahr et al., 2008; Porges et al., 2017; Rmus et al., 2023). These reductions likely reflect impaired astrocyte glutamate-glutamine cycling and weakened glial-neuronal metabolic coupling, rather than simple synaptic pruning, thereby leading to instability in the excitatory-inhibitory balance (Schreiner et al., 2024). GABA reduction, therefore, more likely indicates inefficient astrocytic recycling and diminished interneuron metabolic support, not neuronal loss. Acetylcholine also decreases steadily, with estimates suggesting a rate of approximately 3%–4% per decade, most pronounced in basal forebrain-cortical projections critical for memory encoding (Kuhl et al., 1996; Bohnen et al., 2015). This cholinergic decline stems from cumulative mitochondrial dysfunction and impaired axonal transport efficiency, which weaken cortical modulation and synaptic maintenance.

Although dopamine and serotonin levels remain relatively stable in many older adults, PET reveals subtle but measurable reductions in striatal dopamine that may underlie early motor slowing (Hilker et al., 2005; Berry et al., 2016). Longitudinal imaging suggests that dopaminergic alterations first appear in associative striatal circuits before the motor network declines, indicating that cortical-striatal disconnection emerges before neuronal loss. This network-level dysfunction may serve as an early biomarker of dopaminergic instability and declining cognitive motivational integration.

These shifts are detectable at the preclinical stage, long before cognitive decline becomes measurable (Schreiner et al., 2024). For example, reduced GABA and glutamate in posterior cingulate and medial frontal cortices have been linked to subthreshold impairments in episodic memory and executive function (Riese et al., 2015). Such nonlinear excitatory-inhibitory alterations trace a continuum from temporary adaptation to progressive signaling failure, reflecting how compensatory remodeling gradually collapses as metabolic inefficiency accumulates. AI models that integrate PET, MRS, and MRI data can track these neurochemical trajectories over time, distinguishing between transient stabilization and degenerative signaling failure.

The ability to detect these region-specific neurochemical changes positions AI-based fusion models to uncover network-level imbalances and differentiate healthy from pathological aging. Deep learning models that integrate PET, MRS, and structural MRI measures may identify circuit-specific excitatory-inhibitory imbalances, enabling precision stratification of older adults into resilient versus at-risk groups (Wiesman et al., 2024). AI also offers the capacity to link fluctuations in neurotransmitters to evolving functional connectivity, modeling how neurochemical decline maps onto cognitive trajectories across the lifespan. This approach aligns with the *Compensatory Signaling Hypothesis*, which frames nonlinear neurotransmitter dynamics as adaptive responses that may ultimately fail with progressive metabolic burden—and posits that AI can quantify these transitions to stratify aging trajectories.

### 3.2 Adaptive inhibition transitions into degenerative suppression

Occasional increases in GABA observed in aging likely reflect short-term compensatory inhibition that temporarily stabilizes overactive neural circuits rather than true neurodegeneration (Porges et al., 2021). Over time, however, persistent inhibitory tone suppresses cortical flexibility and slows information processing, marking a mechanistic transition from adaptation to decline (Kuhn et al., 2024). AI models trained on longitudinal multimodal imaging could track this dynamic inflection, identifying the threshold where adaptive compensation transitions to irreversible dysfunction (Huang and Shu, 2025).

### 3.3 Neurodegenerative disorders amplify network-level neurotransmitter collapse

Neurodegenerative disorders amplify and accelerate aging-related changes in neurotransmission. In Alzheimer’s disease, glutamate and acetylcholine decline sharply across cortical and hippocampal regions, while serotonergic binding decreases in the striatum and midbrain (Dhanjal et al., 2014; Matsuoka et al., 2024). MRS evidence also suggests that regional glutamate loss correlates with amyloid and tau accumulation, indicating that excitatory dysfunction may mediate synaptic vulnerability in early stages of AD (Dhanjal et al., 2014). This sequence suggests that glutamate depletion triggers compensatory GABAergic overactivation, ultimately disrupting cortical excitatory drive and network integration. Paradoxically, some datasets identify elevations of GABA in advanced Alzheimer’s, signaling a maladaptive inhibitory overshoot that emerges when early compensation becomes pathological (Schreiner et al., 2024). AI-enabled multimodal modeling could identify the neurochemical inflection points at which protective inhibition transitions to degenerative suppression. A large, multicenter amyloid and tau PET study examined the risk of progression to mild cognitive impairment, as well as cognitive decline rates, among cognitively unimpaired individuals who were amyloid PET-positive and tau PET-positive- in the medial temporal lobe and/or the temporal neocortex (Ossenkoppele et al., 2022). Abnormal amyloid and tau PET findings in cognitively unimpaired individuals are strongly associated with short-term cognitive decline and significantly faster longitudinal global decline (Ossenkoppele et al., 2022). Although these findings are highly clinically relevant, the study was limited by challenges in data pooling and harmonization. Implementing AI can improve data harmonization, especially in multicenter studies, allowing clearer data analysis and further refinement of future findings.

In Parkinson’s disease, striatal dopamine loss is the key feature, but PET and MRS show that cortical cholinergic denervation occurs simultaneously (Hilker et al., 2005; Bohnen et al., 2022). This combined dopaminergic-cholinergic decline contributes not only to motor impairment but also to cognitive deficits (Bohnen et al., 2015). Mechanistically, dopaminergic depletion triggers upregulation in interneurons as an initial attempt to stabilize, which later becomes maladaptive, disrupting corticostriatal balance. MRS on cerebellar function demonstrates that GABA reduction in the cerebellum and substantia nigra accompanies glutamatergic depletion in prefrontal regions, reinforcing the distributed network failure underlying Parkinson’s disease rather than isolated nigrostriatal loss (Piras et al., 2020; Shukla et al., 2023). AI fusion frameworks can characterize these dopaminergic-cholinergic-GABAergic interdependencies, classifying Parkinson’s disease subtypes by the network-level architecture of neurotransmitter systems.

Emerging evidence implicates serotonergic dysregulation in the dorsal raphe and striatal terminals as a contributor to mood disturbances and impulse control symptoms. Late-life depression exhibits reduced serotonin transporter binding in subcortical regions coupled with altered cortical GABA levels, linking monoaminergic and inhibitory dysfunction to affective and cognitive disturbances (Ouchi et al., 2009; Smith et al., 2021a). Importantly, reductions in serotonergic transporter availability within the striatum, thalamus, and midbrain co-occur with glutamate dysregulation in medial frontal regions, suggesting that depression reflects a combined breakdown of inhibitory and excitatory regulation rather than isolated monoaminergic loss (Smith et al., 2021a). Serotonergic deficits also impair astrocytic glutamate clearance, promoting cortical hyperexcitability and microstructural white matter changes detectable via diffusion MRI (Smith). This serotonergic-glutamatergic-astrocytic triad defines depression as a systems-level neurotransmitter failure rather than a single neurotransmitter deficit. AI-driven PET-MRS fusion could disentangle primary serotonergic pathology from secondary excitatory-inhibitory maladaptation, refining diagnostic precision and therapeutic targeting.

Together, these findings suggest a system-level dysregulation in which serotonin, glutamate, and GABA interact to influence both affective symptoms and cognitive decline (Smith et al., 2021b; Schreiner et al., 2024). AI models capable of multimodal integration can quantify these interactions, mapping relationships within neurotransmitter networks to forecast cognitive trajectories. Explainable AI applied to PET-MRS datasets can highlight which combinations, such as posterior cingulate hypoglutamatergia with frontal GABA loss, most strongly predict cognitive decline, enabling mechanistic subtyping for individualized interventions (Wiesman et al., 2024).

Across conditions, converging evidence suggests that reductions in acetylcholine, glutamate, and GABA in the posterior cingulate, frontal cortex, and striatum are strongly correlated with deficits in episodic memory, executive function, and global cognition (Griffith et al., 2008; Riese et al., 2015). These regions form central hubs in large-scale networks such as the default mode, salience, and frontoparietal control systems, and neurotransmitter depletion within these hubs likely destabilizes global network communication. Posterior cingulate hypoglutamatergia, for example, weakens default mode connectivity associated with memory loss in both aging and Alzheimer’s disease, while frontal GABA deficits reduce top-down inhibitory control, thereby underlying executive dysfunction (Riese et al., 2015; Porges et al., 2017). Striatal cholinergic loss further compromises corticostriatal loops essential for cognitive flexibility (Bohnen et al., 2015). Variations in neurotransmitter levels account for a greater share of differences in cognitive performance than structural or metabolic measures alone, highlighting their importance as early, mechanistic biomarkers (Schreiner et al., 2024). AI-driven multivariate models can formalize these relationships, linking neurotransmitter topology directly to cognitive phenotype and enabling individualized disease forecasting.

### 3.4 Neurotransmitter changes track treatment response

Neurotransmitter imaging also provides a framework for evaluating therapeutic response. PET tracers targeting dopaminergic and cholinergic systems have been used to monitor treatment-related modulation in Parkinson’s and Alzheimer’s disease (Brown et al., 2024). Similarly, MRS detects shifts in cortical GABA and glutamate following both pharmacological treatments, such as cholinesterase inhibitors, and non-pharmacological approaches, including cognitive training and exercise (Buard et al., 2022). Longitudinal MRS data show that increases in prefrontal GABA following exercise or cognitive engagement correlate with improvements in executive function, while stable glutamate levels are associated with maintained working memory performance (Buard et al., 2022). These adaptive neurotransmitter shifts signal neurochemical resilience- the restoration of balanced inhibition and excitation that supports cognitive preservation. Deep learning models applied to longitudinal imaging could distinguish transient pharmacodynamic responses from sustained plastic recovery, enhancing sensitivity to therapeutic efficacy and enabling real-time adaptation of treatment strategies.

These neurodegenerative disease observations support a broader reframing of neurodegenerative disorders as failures of neurotransmitter coordination across distributed brain networks. Rather than reflecting isolated losses, conditions such as Alzheimer’s, Parkinson’s, and late-life depression exhibit converging disruptions in glutamatergic, cholinergic, GABAergic, and serotonergic systems that unfold dynamically across space and time. The *Mechanistic Integration Hypothesis* predicts that AI-enabled multimodal neuroimaging can resolve these entangled trajectories, exposing causal relationships between molecular deterioration and functional decline. In parallel, the *Compensatory Signaling Hypothesis* posits that treatment-induced shifts in neurotransmitter levels indicate restored circuit equilibrium, which can be distinguished from transient pharmacological effects through AI-driven longitudinal modeling. Together, these hypotheses point to a future in which mechanistically grounded biomarkers support real-time diagnosis, disease subtyping, and precision therapeutics.

Testing these hypotheses requires longitudinal, multimodal imaging that resolves neurotransmitter, metabolic, and network-level dynamics within individual aging trajectories. Integrating PET-based receptor mapping, MRS-derived metabolite quantification, and fMRI connectivity can expose molecular–circuit dependencies that characterize the transition from adaptation to degeneration. Transformer and graph neural network architectures provide analytical capacity to model these dependencies across modalities, linking receptor density, metabolic state, and network coupling within unified predictive embeddings (Ling et al., 2025; Patel, 2025; Zhang et al., 2025). Cross-modal generative and variational frameworks demonstrate the feasibility of capturing disease-related trajectories via learned latent spaces that link molecular alterations to cognitive decline (Pan and Wang, 2022; Sauty and Durrleman, 2022).

Evaluation of the *Mechanistic Integration Hypothesis* depends on determining whether fused molecular–metabolic–structural representations improve causal inference about neurodegenerative progression beyond unimodal models. Explainable AI approaches such as relevance propagation and attention-based attribution extend this framework and reveal spatial and network loci that contribute most to predictive variance (Nazari et al., 2021b; Khatri and Kwon, 2023). Modeling of neurotransmitter re-equilibration following pharmacologic or behavioral interventions can test the *Compensatory Signaling Hypothesis* and determine whether restored excitatory–inhibitory balance reinstates network stability. Collectively, these frameworks define a conceptual pathway for validating or falsifying proposed mechanisms that link neurotransmitter coupling to neurodegenerative vulnerability.

## 4 Barriers to accurate neurotransmitter mapping

### 4.1 Nonlinear neurotransmitter interactions obscure mechanistic interpretation

Neurotransmitter systems interact dynamically, making single-transmitter models insufficient. Dopaminergic decline often coincides with cortical cholinergic loss, while glutamatergic and GABAergic changes modulate amyloid pathology in Alzheimer’s (Dhanjal et al., 2014; Bohnen et al., 2015). In addition, serotonergic dysregulation frequently overlaps with cholinergic and GABAergic dysfunction in late-life depression, producing complex neurochemical signatures not captured by single pathway models (Smith et al., 2021b). These interdependent patterns reflect network-level compensation, where cross-system receptor signaling reshapes equilibrium under stress. Disruption in one pathway can induce adaptive remodeling in others, blurring the line between pathology and compensation. For example, serotonergic depletion can induce secondary hyperactivity in GABAergic circuits, while dopaminergic deficits trigger the upregulation of cholinergic interneurons as a compensatory attempt to maintain network balance (Ouchi et al., 2009; Bohnen et al., 2022). AI-based multimodal analysis can model these nonlinear dependencies, revealing hidden coupling among neurotransmitters and identifying adaptive versus maladaptive remodeling across circuits.

### 4.2 Measurement distortions undermine quantitative neurotransmitter imaging

PET tracers offer millimeter resolution but face significant challenges, including partial volume effects, uncertainties in the reference region, and competition with endogenous ligands (Sedvall et al., 1990; Tiepolt et al., 2022) (also see Figure 1). Tracer kinetics are further affected by age-dependent shifts in receptor affinity that erode binding precision and reduce signal specificity in older adults (Sedvall et al., 1990). Additionally, there is room for further refinement of glutamate and GABA PET tracers. Glutamate and GABA tracers remain less mature than many dopamine, cholinergic, and serotonin tracers due to their structural complexity. This increases the difficulty of developing specific tracers that can cross the blood–brain barrier (Murrell et al., 2020; Wang et al., 2024). PET/MRS offers robust cortical GABA and glutamate quantification but suffers from large voxel sizes, macromolecule contamination, and field inhomogeneity (Simmonite et al., 2019; Lengu et al., 2021). These issues systematically underestimate metabolite concentrations in regions affected by cortical thinning or cerebrospinal fluid expansion (Dobri et al., 2022). Voxel misalignment across sessions also hinders longitudinal comparison, especially as atrophy alters local tissue composition during aging.

AI reconstruction and harmonization methods now address many of these distortions. Convolutional neural network (CNN)-based super resolution (Song et al., 2020) and generative adversarial network (GAN)-based denoising (Hashimoto et al., 2024) recover spatial detail and improve SNR, while transformer-based harmonization compensates for scanner variability and partial volume loss (Usama et al., 2025). Integrating these AI pipelines directly into acquisition workflows could convert low-fidelity scans into quantitatively interpretable neurochemical maps, narrowing the gap between research-grade and clinical-quality imaging.

Ultra-high-field scanners (≥7T) enhance spatial resolution and spectral separation, but accessibility remains limited to research settings (Platt et al., 2021). Although 7T MRSI enhances signal-to-noise ratio by up to 30%, it introduces B₀ field inhomogeneity and susceptibility distortions that disproportionately affect subcortical voxels (Mahmud et al., 2024). AI-based distortion correction and adaptive coil sensitivity modeling are beginning to mitigate these artifacts, thereby extending ultra-high-field precision to deeper structures (Mahmud et al., 2024). Additionally, the availability of tracers and their short half-lives constrain PET to specialized centers, limiting its large-scale clinical translation (Platt et al., 2021; Brown et al., 2024). AI-driven denoising and harmonization pipelines partially offset these logistical constraints by improving quantitative consistency across sites and scanners, defining a technical pathway toward routine, high-fidelity neurotransmitter mapping (Usama et al., 2025).

#### 4.2.1 Limitations of AI-derived neurotransmitter inference

Although AI-enabled multimodal neuroimaging can improve signal integration and pattern extraction, it cannot directly measure neurotransmitter systems. AI models infer latent patterns from multimodal data, but those inferences depend on the quality, validity, and biological specificity of the underlying measurements (Lundervold and Lundervold, 2019). AI models rely on existing data distributions; when datasets remain limited, noisy, or poorly generalizable, those distributions can propagate uncertainty and bias. Aging and neurodegenerative cohorts amplify this problem because disease heterogeneity, comorbidities, scanner variability, and site effects complicate signal interpretation and reduce model generalizability (Fortin et al., 2018; Pomponio et al., 2020). Rigorous validation frameworks must therefore test whether AI-derived inferences reflect biologically meaningful neurochemical patterns rather than technical or cohort-specific artifacts. External validation across independent cohorts, modality-level harmonization, and inclusion of diverse, representative populations remain essential for ensuring that AI-derived inferences generalize beyond the datasets used for model development (Basha et al., 2026). Future studies that use AI-derived neurotransmitter interpretations should explicitly quantify uncertainty, calibration, bias, and subgroup performance, because unvalidated AI pipelines can amplify measurement limitations rather than correct them. AI-derived outputs should therefore be interpreted as probabilistic model-based inferences, not as direct measurements of neurochemical signaling (Basha et al., 2026).

Positron emission tomography (PET) is affected by scanner and vendor variability, partial volume effects exacerbated by atrophy, differences in reconstruction algorithms, tracer pharmacokinetics, head motion, and resolution variability. Magnetic resonance spectroscopy (MRS) is influenced by vendor-specific acquisition differences, sequence and processing variability, partial volume effects from tissue heterogeneity, macromolecule contamination confounding GABA quantification, B₀ field inhomogeneity, and motion-related frequency offsets. Age-related anatomical changes such as brain atrophy, segmentation errors, and reference region variability further amplify these limitations. Collectively, these artifacts bias neurotransmitter quantification and reduce reproducibility in aging and neurodegenerative cohorts.

### 4.3 Cross-site variability prevents reproducible neurotransmitter mapping

Cross-site variability in scanners, acquisition protocols, and reconstruction pipelines undermines the reproducibility of results. Aging-related atrophy amplifies partial volume errors in PET, while vendor and sequence differences bias MRS quantification (Abad et al., 2014; Bell et al., 2022). Statistical tools like ComBat reduce site effects, and Centiloid scaling provides cross-tracer comparability for amyloid PET; however, equivalent reference frameworks for GABA, glutamate, or dopamine tracers are still lacking (Zounek et al., 2023; Yang et al., 2024).

Deep learning harmonization trained on single cohort data often overfits scanner characteristics, degrading generalizability when deployed on independent datasets (Chen et al., 2020). Model bias is particularly problematic in aging populations, where demographic and genetic diversity affect baseline neurotransmitter levels. Algorithms optimized on homogeneous or younger cohorts may misclassify normal neurochemical aging as pathology, reinforcing inequities in diagnostic accuracy.

To address these issues, multicenter benchmarking initiatives must establish standardized reference datasets and transparent preprocessing protocols. Open-source pipelines modeled after ADNI and the UK Biobank can anchor reproducibility in PET-MRS integration. Combining AI-based harmonization with statistical correction tools, such as ComBat and phantom calibration, will yield reproducible, cross-scanner neurotransmitter metrics that preserve biological variance while controlling technical noise (Table 3). Without such standards, meta-analytic synthesis remains fragmented, and AI-derived biomarkers cannot transition from discovery to clinical validation.

**Table 3:** Data harmonization initiatives and AI relevance.

Initiative	Focus	AI relevance
ADNI (Alzheimer’s disease neuroimaging initiative)	Longitudinal multimodal imaging in AD and MCI	Benchmark dataset for AI models in AD progression
UK Biobank	Population-scale imaging-genetics dataset	Training resource for AI in aging, genetics, multimodal fusion
Human connectome project (HCP)	High-resolution structural and functional imaging	Ground truth for AI models linking structure, function, neurotransmission
ENIGMA consortium	Global collaboration on multimodal neuroimaging datasets	Meta-analysis integration and federated learning platforms
ComBat harmonization	Statistical correction of site and scanner effects	Preprocessing step improving AI model reproducibility
Centiloid scaling	Standardization of amyloid PET across tracers/protocols	Facilitates AI models trained on amyloid PET harmonized scales
Phantom-based corrections	Scanner calibration using standardized phantoms	Provides calibration data for training harmonization networks
Deep learning harmonizers	AI-driven cross-site harmonization and bias correction	Directly applies deep learning to overcome site effects

Major initiatives include ADNI, UK Biobank, Human Connectome Project, and ENIGMA, each providing large-scale multimodal imaging resources. Statistical harmonization approaches such as ComBat and Centiloid scaling improve reproducibility, while phantom-based corrections and deep learning harmonizers address scanner- and site-specific biases. Together, these resources and methods establish the foundation for AI-driven reproducibility in neurotransmitter mapping.

### 4.4 Regulatory and clinical barriers limit deployment of AI neurotransmitter biomarkers

Even as technical challenges are mitigated, regulatory obstacles slow adoption. AI-driven imaging biomarkers fall under FDA and CE medical device frameworks, including 510(k), *de novo*, and Software as a Medical Device (SaMD) classifications (Singh et al., 2022; Yearley et al., 2023). Most current neuroimaging algorithms fail to meet these thresholds because they lack multicenter validation, standardized performance metrics, and transparent versioning (Pemberton et al., 2021; Khunte et al., 2023). Additional barriers include limited clinician interpretability of AI outputs, cross-border data privacy restrictions, and uncertain reimbursement mechanisms (Goodkin et al., 2019). Clinicians often resist the adoption of “black box” systems, emphasizing the need for explainable AI (XAI) architectures that visualize saliency maps, expose decision logic, and quantify predictive uncertainty (Wiesman et al., 2024). Regulatory agencies are beginning to integrate these interpretability criteria, but formal standards for clinical grader XAI remain in development. As a result, most AI methods remain confined to proof-of-concept implementations rather than being deployed as diagnostic tools. Bridging this gap demands shared validation datasets, transparent model governance, and performance reporting aligned with clinical trial endpoints (Singh et al., 2022). Without these steps, AI-enabled neurotransmitter biomarkers risk stagnating with academic research rather than evolving into regulatory-grade instruments for precision diagnostics and therapy monitoring.

The barriers outlined in this section define the experimental and translational constraints that limit the empirical testing of the Coupling Pattern and Mechanistic Integration Hypotheses. Nonlinear neurotransmitter interactions create signal overlap that current imaging modalities cannot fully resolve, producing confounds that distort excitatory–inhibitory coupling and obscure mechanistic interpretation (Bell et al., 2022; Yang et al., 2024). PET and MRS quantification errors, including partial volume effects, voxel misalignment, and macromolecule contamination, reduce reproducibility and degrade the precision needed to model neurotransmitter dynamics in aging populations (Thabit et al., 2022). Cross-site differences in scanner design, tracer kinetics, and acquisition protocols further erode comparability across cohorts, a challenge only partially mitigated by ComBat harmonization and phantom-based calibration (Wakasugi et al., 2024; Lin et al., 2025).

AI-based reconstruction and harmonization pipelines now begin to address these distortions. GAN and transformer architectures recover spatial fidelity and suppress scanner bias, while deep harmonization networks achieve consistency comparable to within-site test–retest benchmarks (Thabit et al., 2022; Yang et al., 2024). Integrating these correction methods directly into acquisition workflows will enable mechanistic inference at a scale suitable for hypothesis testing. Nonetheless, reproducibility depends on transparent datasets and regulatory oversight. Without multicenter validation and explainable AI standards, cross-modal fusion cannot transition from research to clinical biomarker development. Overcoming these nonlinear, technical, and regulatory barriers will allow AI frameworks to capture authentic neurotransmitter coupling patterns and reveal the mechanistic pathways that govern neurochemical aging (Bell et al., 2022; Lin et al., 2025).

Testing these hypotheses within this framework requires designs that quantify the interaction between technical and biological variance across imaging modalities. AI harmonization models can simulate controlled perturbations in scanner bias (Thabit et al., 2022; Wakasugi et al., 2024; Ying et al., 2024), voxel composition (Abad et al., 2014), and tracer kinetics (Yang et al., 2024) to determine whether neurotransmitter coupling patterns persist after eliminating acquisition artifacts. This approach converts harmonization from a preprocessing step into an experimental variable that reveals which correlations represent authentic neurobiology rather than technical noise (Bell et al., 2021, 2022). Mechanistic integration can be tested by embedding harmonized PET, MRS, and MRI datasets into cross-modal graph neural networks that model neurotransmitter covariance across cortical and subcortical systems. If these models retain predictive stability when trained on harmonized but demographically heterogeneous cohorts, the results demonstrate biological generalizability rather than site-specific bias (Matheson et al., 2025; Yang et al., 2024). Finally, cross-validation using synthetic data generated through generative AI simulators can benchmark the fidelity of harmonization models, defining quantitative thresholds for when signal correction preserves mechanistic integrity (Chen et al., 2020; Thabit et al., 2022).

### 4.5 Relationship to normative modeling, virtual brains, and digital twins

AI multimodal neurotransmitter mapping can add an analytical layer within broad computational neuroscience frameworks such as normative modeling, brain network models, virtual brains, and digital twins. Normative modeling provides reference distributions that quantify individual deviations in brain structure and function, allowing the identification of abnormal aging trajectories and changes indicative of pathology or disease (Ge et al., 2024). Earlier applications of normative modeling focused primarily on structural variations of brain development and aging (Ziegler et al., 2014; Wolfers et al., 2018). It is also a promising novel approach to investigate deviations in the brains of those diagnosed with neuropsychiatric disorders (Marquand et al., 2019). Multimodal fusion of PET and MRS can augment normative frameworks by incorporating neurotransmitter-specific variables, thereby enabling alterations to be interpreted at a neurochemical scale (Marquand et al., 2016; Liu et al., 2022).

Virtual brain platforms are brain network models that describe and predict dynamic interactions between neurons and predict network behavior under varying physiological conditions (Leon et al., 2013). One of the state-of-the-art models constrains regional heterogeneity and fine-tunes regional dynamics according to measures of inhibitory and excitatory gene expression (Deco et al., 2021). This enables the model to replicate the spatiotemporal structure of empirical estimates of functional connectivity with greater accuracy than models restricted to global gene expression profiles or MRI-derived myelination estimates. Data-driven models have also been developed. One of these models infers neural mass models from functional data that represent regional and individualized dynamics while maintaining known network structures (Sip et al., 2023). When this approach is applied to human resting-state fMRI data, the underlying dynamics can be represented as noisy fluctuations around a single, fixed point. The model was able to reproduce features of original, known datasets and elicit both regional and subject-specific parameters that strongly correlate with known, original parameters. AI can enhance these models by providing specific, quantifiable, evidence-based parameters for synaptic gain, balance between excitation and inhibition, and neuro-modulatory tone. This provision can improve generalizability and predictive validity. Instead of replacing network modeling, multimodal neurotransmitter mapping can help better link molecular changes to alterations within a broader system.

Digital twin frameworks are virtual representations of physical objects, systems, or processes that are updated with data to match the life cycle of their physical counterparts (Fekonja et al., 2024). These systems have been successfully implemented in architecture, manufacturing, design, city planning, and more (Rosen et al., 2015; Al-Sehrawy and Kumar, 2021; Helbing and Sánchez-Vaquerizo, 2023; Yossef Ravid and Aharon-Gutman, 2023; Fekonja et al., 2024). Digital twins can be used in healthcare, as they can represent anatomical structures and simulate physiological dynamics, particularly in neuroscience (Fekonja et al., 2024). Digital twins create individualized, longitudinal representations of brain states that serve as predictors and aid in intervention planning (Hashemi et al., 2026). There are numerous challenges associated with constructing a digital twin, particularly of the brain, due to its complexity. Although challenges must be addressed before the use of digital brain twins in concrete applications, digital brain research has already begun in both research and real-world settings (Amunts et al., 2024). Digital brain research integrates data models, theory, methods, and computational technologies for research and development efforts within the framework of the Human Brain Project (HBP) (Amunts et al., 2024). The HBP is a large-scale brain research project that began in 2013 and concluded in 2023, which played a major role in making digital brain research more collaborative, reproducible, and ethical (Amunts et al., 2022, 2024). AI-enabled multimodal fusion can potentially improve the accuracy of digital twin frameworks and contribute to digital brain research by generating individualized neurochemical profiles that can be updated and further personalized over time. This allows refinement of predictions of disease progression and treatment response, which is particularly important in aging and neurodegenerative diseases, as changes typically occur along a nonlinear trajectory.

While the above neuroscience frameworks can generate individualized predictions based on reference distributions and network dynamics, AI-enabled multimodal fusion can enhance the predictability and clinical translation of these networks by providing refined mechanistic inputs. This relationship can link molecular-level pathology to network irregularities and tangible clinical outcomes in the neuroscience of aging and neurodegenerative disease.

## 5 AI drives mechanistic and translational advances

### 5.1 AI enhances image quality through reconstruction and denoising

The ability to distinguish biological variance from technical noise defines the experimental ground on which advanced AI systems now operate. These systems extend harmonization into active reconstruction—recovering, refining, and predicting neurochemical patterns that underlie aging and neurodegeneration. Deep learning methods have transformed image preprocessing for neurotransmitter mapping. Convolutional neural networks (CNNs), such as 3D Dense-U-Net, can denoise PET and MRS data, recovering signals originally lost to scanner noise and motion artifacts, particularly in older participants (Lee et al., 2019; Odusami et al., 2023a). CNNs are able to analyze, reconstruct, and impute PET signals such as tau pathology with a correlation coefficient of 0.79 +/− 0.06 and a mean percentage of error of 8.24% (Lee et al., 2019). Both CNN and cGAN frameworks denoise amyloid PET data, reducing the mean relative error to 2%, while also strengthening the association between PET signals and cognitive presentations by up to 67% (Liu et al., 2018). Generative adversarial architectures such as 3D-StyleGAN can simulate amyloid dynamics with low reconstruction error (0.08) and high discriminability (Bossa et al., 2024). Additionally, GANs enable super-resolution, allowing low-dose and shorter-duration scans to approximate high-dose PET acquisitions, thereby reducing radiation exposure (Liu et al., 2021). Pareto-optimized VGG19 models further enhance MRI-PET fusion and increase structural similarity for Alzheimer’s disease diagnosis and classification (Odusami et al., 2023a). Transformer-based models further expand these gains by learning long-range dependencies across imaging volumes, thereby enabling reconstructions with improved structural fidelity (Khatri and Kwon, 2023; Ling et al., 2025; Zhang et al., 2025). Forecasting models integrate CNNs with long short-term memory (LSTM) to predict future PET metabolism with high accuracy (MAE = 0.031), thereby reducing the need for repeated scanning in older adults (Doering et al., 2024). All of these approaches enhance sensitivity and reproducibility, especially in aging cohorts that face barriers such as motion, low signal-to-noise ratio, and limited tolerance for long acquisitions (Lee et al., 2019; Bossa et al., 2024).

It is important to note that the use of AI comes with its own set of limitations. The inclusion of high-dimensional multimodal data introduces a risk of overfitting, the phenomenon of performing almost perfectly in a small set of data while being unable to generalize to a larger population, especially in aging and neurodegenerative studies where sample sizes remain limited (Varoquaux et al., 2017; Varoquaux, 2018; Avberšek and Repovš, 2022). Models may also be unable to generalize independent datasets, which introduces the risk of including cohort biases and site-specific variability in the data (Varoquaux, 2018; Suleman et al., 2025). To address these limitations, it is essential to implement rigorous validation techniques and increase effort to preserve generalizability, ensuring that these models reflect true neurological changes rather than statistical overfitting. Numerous techniques have been developed to reduce overfitting. These techniques are referred to as regularization methods and aim to reduce the generalization error rather than training errors. These models typically involve constraints or penalties on the model’s internal parameters (Goodfellow et al., 2014).

### 5.2 AI integrates multimodal data to reveal neurochemical interactions

AI demonstrates an exceptional ability to integrate heterogeneous imaging modalities to capture interactions between neurotransmitter systems (Figure 2). Feature-level fusion models, such as modified RESNet18, are able to combine PET-derived receptor density maps with MRS-based neurochemical concentrations, revealing coupled dynamics such as dopaminergic decline correlating with glutamatergic hyperexcitability and improving diagnostic accuracy for early Alzheimer’s disease detection (Odusami et al., 2023a, 2023b; Martinez-Murcia et al., 2024). Attention-driven fusion frameworks that integrate MRI and PET data using hybrid deep learning and support vector machines have achieved classification accuracies up to 98.5%, highlighting the effectiveness of multimodal imaging combined with attention mechanisms (Paduvilan et al., 2025). Complementary approaches using multimodal vision transformers with cross-attention modules fuse sMRI and fMRI, yielding high predictive performance while enabling interpretable modeling of brain connectivity patterns (Bi et al., 2024). Recent evidence further emphasizes that integrating MRI, PET, and fMRI through AI-powered architectures holds promise for precision diagnostics, though specific quantitative benchmarks vary across datasets (Huang and Shu, 2025; Paduvilan et al., 2025). Decision-level fusion leverages complementary information by weighing modality-specific predictions for tasks involving classification or prognosis tasks. This approach can reveal relationships that are otherwise hidden by unimodal analyses, between neurotransmitter signaling and functional network breakdown. Clinical-imaging fusion combines MRI, FDG-PET, and clinical features using SVMs or random forests, resulting in a higher area under the curve (0.89) than any single modality and offering a more robust prediction of progression from mild cognitive impairment to Alzheimer’s disease (Samper-Gonzalez et al., 2019). Integrating multi-scale data at molecular, metabolic, and structural levels with fusion methods can establish a mechanistic bridge between neurochemical changes and circuit-level dysfunction. These representations establish the neurochemical and structural foundations that predictive and mechanistic models can utilize to forecast trajectories and disease progression.

### 5.3 AI predicts disease trajectories from neurotransmitter maps

AI can also project disease trajectories from early imaging signatures. Recurrent neural networks and hybrid mechanistic AI models utilize neurotransmitter maps to predict the progression of Alzheimer’s disease from mild cognitive impairment or to identify prodromal Parkinsonian syndromes (Yu et al., 2023). By incorporating declines in neurotransmitter levels into longitudinal models, AI can generate dynamic biomarkers that go beyond static group comparisons. Three-dimensional CNNs predict progression from mild cognitive impairment to Alzheimer’s disease with accuracies ranging from 72% to 92% (Choi and Jin, 2018; Prats-Climent et al., 2022; Khatri and Kwon, 2023). Mechanistic-informed architectures integrate domain knowledge of processes such as dopaminergic pathways and the glutamate-GABA balance with data-driven features, enabling the development of biologically interpretable predictors. Personalized multifactorial modeling combines PET with tau measurements, amyloid-beta, glucose metabolism, and structural features from MRI to estimate neurotransmitter fluctuations and changes across disease stages (Khan et al., 2021). These models and the predictors they produce can stratify patients by neurotransmitter vulnerability, informing individualized risk assessment and treatment plans.

### 5.4 Explainable AI improves transparency and trust

Implementation in the clinical setting not only requires accurate predictions but also transparent reasoning. Explainable AI techniques show which brain regions and neurotransmitter features drive model outputs. Saliency maps highlight receptor-enriched regions that contribute to classification, and attention-weighted models quantify prediction uncertainty (Dentamaro et al., 2024). This feature set increases trust in AI outputs and strengthens the refinement of mechanistic hypotheses. Uncertainty quantification constrains overfitting because its confidence maps explicitly flag low-confidence predictions, a function that becomes especially critical when modeling heterogeneous aging populations (Dentamaro et al., 2024; Ling et al., 2025), as depicted in Table 4.

**Table 4:** Explainable AI (XAI) methods in neuroimaging.

XAI method	Strengths	Weaknesses	Application in neuroimaging
Saliency maps	Highlights spatial regions driving predictions	Prone to noise and instability	Highlighting neurotransmitter-enriched brain regions
Layer-wise relevance propagation (LRP)	Provides fine-grained feature attribution	Computationally intensive	Explaining voxel-level importance in PET/MRS models
Grad-CAM (gradient-weighted class activation mapping)	Intuitive heatmaps for model interpretability	Resolution limited to final layers	Identifying cortical/subcortical areas relevant for classification
Attention mechanisms	Captures long-range dependencies in data	May not always align with biological plausibility	Improving interpretability in multimodal fusion models
SHAP (Shapley additive explanations)	Quantifies feature contribution with strong theoretical basis	Complex, requires careful interpretation	Understanding contributions of multimodal features
LIME (Local interpretable model-agnostic explanations)	Simple, model-agnostic, easy to implement	Approximate, sometimes inconsistent explanations	Local interpretability in small-cohort studies
Uncertainty quantification	Detects low-confidence predictions, prevents overfitting	Does not explain *why* uncertainty occurs	Flagging unreliable predictions in aging populations

Saliency maps, LRP, Grad-CAM, attention mechanisms, SHAP, LIME, and uncertainty quantification offer complementary strategies for interpreting AI models in neuroimaging (De Santi et al., 2022; Prats-Climent et al., 2022) (Table 4). Layer-wise propagation (LRP) enables voxel-wise and concept-level interpretability for PET and SPECT models, facilitating the identification of striatal and extrastriatal dopamine transporter distribution and patterns relevant to Parkinsonism diagnosis and classification (Nazari et al., 2021a, 2021b). Concept relevance propagation (CRP) enhances insignia by mapping learned representations to anatomical MRI concepts, clarifying how deep models can encode atrophy and metabolic disruption (Tinauer et al., 2024). Occlusion and feature-importance analyses can quantify a fusion model’s dependence on specific imaging channels and cortical regions, thereby improving confidence in its reliance on meaningful inputs (Lee et al., 2019; Manasova et al., 2024). Each method offers distinct strengths and weaknesses, from voxel-level feature attribution to quantifying prediction uncertainty. Applications in neurotransmitter mapping include highlighting receptor-enriched regions, explaining classification outputs, and flagging unreliable predictions in heterogeneous aging cohorts.

### 5.5 AI advances move toward mechanistic translation

Applications from related domains show how AI-driven imaging can advance biomarker discovery. Oncology radiomics has established workflows for multimodal feature extraction, harmonization, and predictive modeling that inform neuroimaging pipelines (Manasova et al., 2024). Pilot applications of similar methods in neurotransmitter mapping demonstrate feasibility. AI-enhanced PET detects early dopaminergic deficits in cohorts at risk of Parkinson’s disease. Fusion of MRS and PET data also identifies glutamate–dopamine interactions that predict cognitive decline (Martinez-Murcia et al., 2024). These examples highlight the potential, promise, and translational hurdles of adapting AI-enabled frameworks to aging neuroscience.

AI-driven imaging workflows test the *Mechanistic Integration Hypothesis* when models reconstruct neurotransmitter network reorganization across disease stages and treatment conditions. Multimodal fusion and predictive modeling reveal whether molecular, metabolic, and structural features converge into coherent signatures that anticipate transitions from healthy to pathological aging. These models also directly examine the *Compensatory Signaling Hypothesis* as they track therapy-induced neurotransmitter shifts and measure whether those changes stabilize network function or trigger transient adaptations. Integrating explainable AI with longitudinal analyses enables investigators to connect receptor-level dynamics with circuit stabilization, thereby defining when plasticity restores equilibrium rather than accelerating degeneration. Collectively, these strategies transform mechanistic hypotheses into testable, computationally grounded frameworks that unite discovery with clinical translation.

To test these hypotheses, multimodal AI models can integrate PET, MRS, and MRI data into shared latent spaces that represent neurotransmitter coupling across brain networks (Samper-Gonzalez et al., 2019; Pan and Wang, 2022; Sauty and Durrleman, 2022; Odusami et al., 2023b; Subramanyam Rallabandi and Seetharaman, 2023; Yu et al., 2023; Patel, 2025). Each model would undergo training on harmonized datasets spanning aging and neurodegenerative populations, supported by harmonization frameworks that control scanner and site bias while preserving biological variance (Rahim et al., 2015; Pena et al., 2019; Bossa et al., 2024; Jeong et al., 2024; Mohamed Amine and Mourad, 2025). Longitudinal modeling approaches could track how neurotransmitter distributions evolve over time, adapting temporal dynamics from hybrid CNN-LSTM and variational autoencoder architectures that capture disease progression (Sauty and Durrleman, 2022; Doering et al., 2024; Manasova et al., 2024; Martinez-Murcia et al., 2024; Tinauer et al., 2024). Transformer and graph neural network architectures can map dependencies that quantify how receptor density, neurotransmitter concentration, and regional connectivity co-vary during stabilization or decline (Khatri and Kwon, 2023; Bapat et al., 2024; Dan et al., 2025; Hagos et al., 2025; Ling et al., 2025; Zhang et al., 2025). Explainable AI techniques such as layer-wise relevance propagation, Grad-CAM, and concept relevance propagation reveal which molecular features drive recovery versus degeneration (Luckett et al., 2019; Nazari et al., 2021a, 2021b; De Santi et al., 2022; Prats-Climent et al., 2022; Dentamaro et al., 2024; Vázquez-García et al., 2024; Patel, 2025). Validation requires linking these mechanistic representations to observe cognitive and motor performance, thereby establishing a causal alignment between molecular adaptation and behavioral outcomes (Doering et al., 2024; Martinez-Murcia et al., 2024; Vázquez-García et al., 2024).

## 6 Ethical, collaborative, and translational requirements for AI neuroimaging

### 6.1 Equity and ethical governance anchor responsible AI neuroimaging

AI-enabled neurotransmitter mapping depends on the fidelity of its underlying data. The *Coupling Pattern Hypothesis* assumes that neurotransmitter relationships represent biological reality rather than demographic artifacts, yet biased datasets can distort those relationships. Underrepresentation of racial, ethnic, and socioeconomic groups introduces algorithmic drift that may mischaracterize normative coupling trajectories across aging populations. Fairness-aware learning frameworks and federated training pipelines (Tariq et al., 2020) must therefore serve as ethical instruments that preserve model validity across different cohorts. Equitable data inclusion is not only a moral imperative but a mechanistic safeguard, ensuring that cross-system relationships generalize across diverse neurobiologies. Patient advocacy groups and community stakeholders must participate in defining inclusion standards, establishing governance that aligns ethical oversight with the *Mechanistic Integration Hypothesis*. Ethical AI in neuroscience, therefore, extends beyond privacy and consent—it requires epistemic equity to prevent mechanistic misrepresentation of the human brain (Fanijo et al., 2023).

### 6.2 Multidisciplinary collaboration enables mechanistic and clinical integration

Testing and translation of the three hypotheses rely on coordinated expertise spanning computation, neurobiology, and clinical practice. Mechanistic Integration requires that data scientists, radiochemists, and clinicians converge to design models that fuse receptor mapping, metabolic dynamics, and cognitive outcomes into a coherent neurobiological framework. The *Compensatory Signaling Hypothesis* similarly depends on longitudinal validation embedded in clinical workflows, in which pharmacologic and behavioral interventions can reveal adaptive network recalibration. Collaborative infrastructures such as ADNI (Jack et al., 2011, 2018) exemplify the translational scaffolding needed for such efforts. These initiatives not only harmonize data pipelines but also institutionalize dialogue between algorithm developers and end users, ensuring that model interpretability aligns with clinical reasoning. Mechanistic transparency thus becomes a shared language through which discovery science transitions into actionable diagnostics.

### 6.3 A translational pipeline from mechanism to clinical deployment

**Figure 3:**
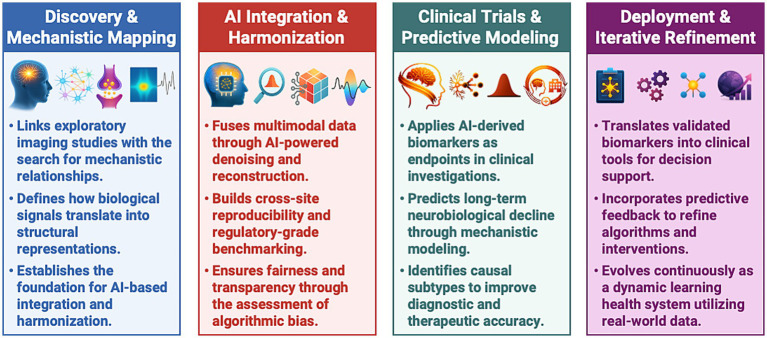
AI-enabled neurobiological research and translation pipeline: from discovery to deployment. This figure presents a four-phase translational sequence capturing the integration of artificial intelligence into neurobiological investigation and clinical application. *Discovery & Mechanistic Mapping* initiates the process by linking exploratory imaging to mechanistic relationships—illustrated by a brain profile, a synaptic cleft, a multicolor network, and a waveform—each representing signal detection, neural transmission, pattern mapping, and biological encoding. *AI Integration & Harmonization* advances this by fusing multimodal data through denoising, benchmarking, and bias assessment, conveyed through a brain-modeling interface, algorithmic connectors, a cube for cross-site comparison, and a harmonized waveform. *Clinical Trials & Predictive Modeling* translates these outputs into trial-ready biomarkers, supporting long-term modeling and subtype identification, with icons reflecting cognitive endpoints, mechanistic linkage, population analysis, and statistical inference. Finally, *Deployment & Iterative Refinement* demonstrates how validated tools transition into clinical environments, update through predictive feedback, and evolve into learning systems—represented by an implementation dashboard, an adaptive algorithm, a system architecture, and a continuous signal loop. Together, the panels depict a self-refining AI pipeline that moves seamlessly from foundational discovery to real-world deployment.

The three hypotheses converge into a translational pathway that links mechanistic fidelity to regulatory readiness (see Figure 3). The *Coupling Pattern Hypothesis* grounds reproducibility metrics in the stability of cross-modal representations; the *Mechanistic Integration Hypothesis* defines how multimodal biomarkers can achieve causal credibility; and the *Compensatory Signaling Hypothesis* sets the framework for real-time therapeutic monitoring. Harmonizing datasets across ADNI, UK Biobank, and the Human Connectome Project enables systematic validation of these mechanisms at scale, while explainable AI methods ensure that inferred neurotransmitter dynamics remain interpretable to clinicians and regulators alike (Khunte et al., 2023). Clinical decision-support systems can then stratify patients by neurotransmitter vulnerability, predict treatment trajectories, and generate biomarkers that meet FDA and CE standards for Software as a Medical Device (Busis et al., 2024). Translation therefore depends on aligning mechanistic rigor with ethical accountability—a dual foundation ensuring that AI-driven neuroimaging advances precision medicine without compromising trust.

### 6.4 Ethical validation of mechanistic models

Testing the ethical and translational robustness of the three hypotheses requires simulation and audit frameworks rather than traditional experiments. Federated learning environments can evaluate whether AI models maintain coupling stability and predictive integrity when trained on demographically heterogeneous cohorts, confirming that the *Coupling Pattern Hypothesis* holds across populations. Model audit pipelines that quantify transparency, uncertainty, and interpretability test the *Mechanistic Integration Hypothesis* by ensuring that mechanistic outputs remain explainable and reproducible under regulatory scrutiny. Clinical shadow trials—parallel observational deployments of AI decision-support systems—can evaluate the *Compensatory Signaling Hypothesis* by monitoring whether real-world feedback from clinicians and patients aligns with predicted dynamics of neurotransmitters. These ethical validation mechanisms transform translation itself into an empirical process, ensuring that mechanistic discovery, fairness, and clinical accountability advance in parallel.

## 7 Conclusion

AI-enabled multimodal neuroimaging offers a transformative framework for mapping neurotransmitter systems in normal aging and age-related disease. Although PET, MRS, and molecular or fluorescence-based imaging each provide unique strengths and access to neurotransmitter systems *in vivo*, limitations in sensitivity, specificity, and reproducibility limit their translational value. These approaches produce constrained and fragmented views that cannot capture the multimodal complexity present in neurotransmitter systems in healthy aging or in neurodegenerative and psychiatric disease. The use of artificial intelligence can potentially solve the problems posed by today’s imaging modalities. By harmonizing and fusing these modalities, AI methods enhance signal quality, correct artifacts, and integrate neurochemical, metabolic, and structural features into coherent maps of excitatory–inhibitory balance and transmitter-specific vulnerabilities. These models enable imaging to transition from a purely descriptive source to one capable of biomarker discovery, mechanistic hypothesis testing, and predictive modeling of disease trajectories at the individual patient level. To effectively and ethically integrate AI into both research and clinical practice, it is critical to invest in diverse, large-scale datasets, federated learning structures, and collaboration involving clinicians, imaging scientists, AI experts, and patient stakeholders. It is also essential to implement regulatory clarity and reimbursement structures to ensure equitable adoption in the clinical setting. The field must commit to coordinated investment across multiple disciplines to transform neuroimaging from unimodal, fragmented snapshots into a precise, predictive tool that can be utosed in both understand and treat age-related neurological disorders. The three hypotheses outlined in this framework, Coupling Pattern, Mechanistic Integration, and Compensatory Signaling, form the foundation of this transformation (Figure 3). The *Coupling Pattern Hypothesis* establishes the foundation by mapping how neurotransmitter systems co-vary at the molecular and circuit levels. The *Mechanistic Integration Hypothesis* builds on this by linking multimodal fusion to causal inference and longitudinal prediction, demonstrating how AI-driven harmonization converts complexity into mechanistic insight. The *Compensatory Signaling Hypothesis* closes the loop, defining how neurotransmitter re-equilibration reflects adaptive or maladaptive recovery within aging networks. Together, these hypotheses describe a unified vision for AI-enabled neuroimaging: one that moves beyond representation to explanation, beyond prediction to intervention. Achieving this vision requires ethical governance, open collaboration, and continued investment in multimodal infrastructure. When these conditions align, AI will not only map the neurotransmitter architecture of aging but also reveal the mechanistic code that underlies resilience and decline.
